# Redox Regulation of Starch Metabolism

**DOI:** 10.3389/fpls.2018.01344

**Published:** 2018-09-21

**Authors:** Katsiaryna Skryhan, Libero Gurrieri, Francesca Sparla, Paolo Trost, Andreas Blennow

**Affiliations:** ^1^Department of Plant and Environmental Sciences, University of Copenhagen, Frederiksberg, Denmark; ^2^Department of Pharmacy and Biotechnology – FaBiT, University of Bologna, Bologna, Italy

**Keywords:** redox regulation, starch, thioredoxins, NTRC, diurnal regulation

## Abstract

Metabolism of starch is a major biological integrator of plant growth supporting nocturnal energy dynamics by transitory starch degradation as well as periods of dormancy, re-growth, and reproduction by utilization of storage starch. Especially, the extraordinarily well-tuned and coordinated rate of transient starch biosynthesis and degradation suggests the presence of very sophisticated regulatory mechanisms. Together with the circadian clock, land plants (being autotrophic and sessile organisms) need to monitor, sense, and recognize the photosynthetic rate, soil mineral availability as well as various abiotic and biotic stress factors. Currently it is widely accepted that post-translational modifications are the main way by which the diel periodic activity of enzymes of transient starch metabolism are regulated. Among these mechanisms, thiol-based redox regulation is suggested to be of fundamental importance and in chloroplasts, thioredoxins (Trx) are tightly linked up to photosynthesis and mediate light/dark regulation of metabolism. Also, light independent NADP-thioredoxin reductase C (NTRC) plays a major role in reactive oxygen species scavenging. Moreover, Trx and NTRC systems are interconnected at several levels and strongly influence each other. Most enzymes involved in starch metabolism are demonstrated to be redox-sensitive *in vitro*. However, to what extent their redox sensitivity is physiologically relevant in synchronizing starch metabolism with photosynthesis, heterotrophic energy demands, and oxidative protection is still unclear. For example, many hydrolases are activated under reducing (light) conditions and the strict separation between light and dark metabolic pathways is now challenged by data suggesting degradation of starch during the light period.

## Starch Metabolism: Enzymatic Machinery and Regulation

Plants accumulate starch as both a transient and long-term carbohydrate reserve. As a result, starch metabolism must be adjusted to provide ample carbon supply in response to many physiological demands mainly related to nocturnal, stress, and germination events (Stitt and Zeeman, 2012; Lloyd and Kossmann, 2015; Hedhly et al., 2016; Zanella et al., 2016; Pirone et al., 2017; Thalmann and Santelia, 2017). Coordinated metabolic flux among starch biosynthetic enzymes can also permit correct structuring of the starch granule irrespectively of carbon flow (Blennow and Svensson, 2010; Glaring et al., 2012; Pfister and Zeeman, 2016; Botticella et al., 2018).

**FIGURE 1:**
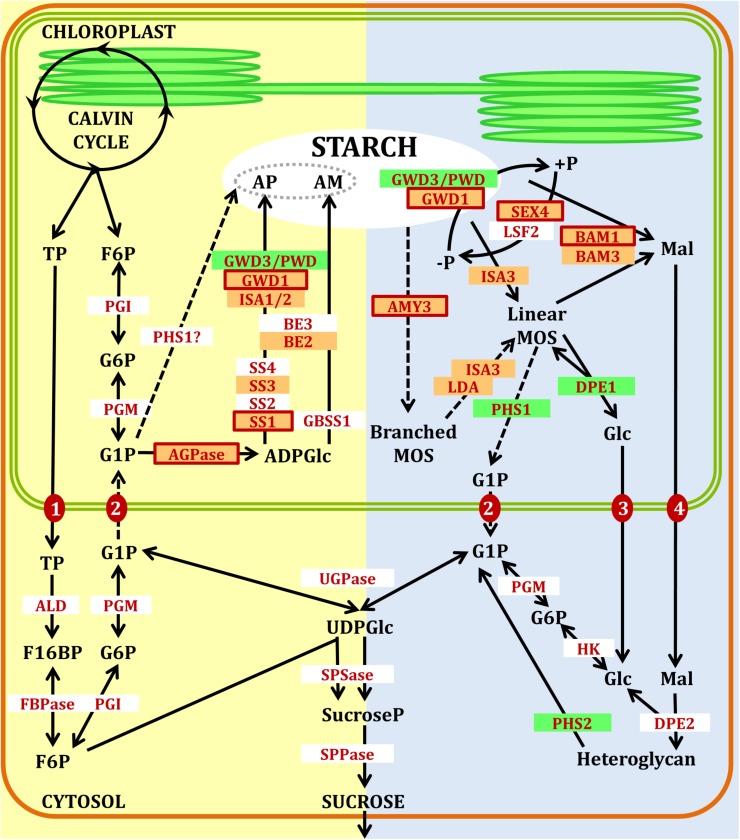
Pathways of starch synthesis (yellow) and degradation (blue) in Arabidopsis leaves. Orange boxes with red outlines represent well confirmed redox regulated enzymes. Orange boxes with no outlines represent suggested redox regulated enzymes. Green boxes represent redox tolerant enzymes. AM, amylose; AP, amylopectin; TP, triosephosphates; F6P, fructose-6-phosphate; G6P, glucose-6-phosphate; G1P, glucose-1-phosphate; ADPGlc, ADP-glucose; MOS, maltooligosaccharides; Mal, maltose; Glc, glucose; F16BP, fructose-1,6-bisphosphate; UDPGlc, UDP-glucose; SucroseP, sucrose-phosphate; ALD, aldolase; FBPase, fructose-1,6-bisphosphatase; PGI, phosphoglucose isomerase; PGM, phosphoglucomutase; AGPase, ADP-glucose pyrophosphorylase; SS, soluble starch synthase isoforms; GBSS, granule-bound starch synthase; BE, branching enzyme; ISA, isoamylase; LDA, limit dextrinase; PHS, α-glucan phosphorylase; GWD1, glucan water dikinase; PWD, phosphoglucan, water dikinase; SEX4 and LSF2, phosphoglucan phosphatases; BAM, β-amylase; AMY, α-amylase; DPE, disproportionating enzyme; FBPase, fructose-1,6-bisphosphatase; UGPase, UDP-glucose pyrophosphorylase; SPSase, sucrose-phosphate synthase; SPPase, sucrose phosphate phosphatase; HK, hexokinase. Transporters are shown as red filled circles: (1) triose-phosphate/phosphate translocator (TPT); (2), G1P translocator; (3) plastidial glucose transporter (pGLT); (4) maltose transporter (MEX1). Dashed arrows represent minor or possible routes. Figure inspired from Stitt and Zeeman (2012).

The reactions of starch metabolism are catalyzed by a series of enzymes (**Figure 1**), mainly regulated to account for transitory starch biosynthesis during active photosynthesis and mobilization at night (MacNeill et al., 2017). These reactions can be functionally classified as synthetic or degradative reactions. However, these should not be considered as strictly alternative reactions as transitory starch degradation is suggested to occur simultaneously with starch synthesis under long day conditions (Fernandez et al., 2017).

To allow tight communication between energy demands at a multitude of conditional situations, starch metabolism is regulated at several levels (Kötting et al., 2010; Stitt and Zeeman, 2012; MacNeill et al., 2017). The rate of both starch biosynthesis as well as starch degradation correlates with the anticipated length of the night directly or reversibly (Smith and Stitt, 2007), providing evidence of a tight circadian control (Graf et al., 2010). Moreover, simultaneously monitoring photosynthetic rate, soil minerals availability, various abiotic and biotic stress factors requires precise mechanisms adjusting the rate of transient starch turnover in response to these stimuli (Thalmann and Santelia, 2017) and both transcriptional and post-translational levels are important (Geigenberger, 2011; Stitt and Zeeman, 2012; Geigenberger and Fernie, 2014; Santelia et al., 2015; Mahlow et al., 2016; MacNeill et al., 2017).

Regulation at the transcriptional level provides a mid- to long-term adjustment of starch turnover (Stitt and Zeeman, 2012) while post-translational modifications are currently accepted to be the main way by which the diel periodic activity of enzymes of transient starch metabolism are regulated (Kötting et al., 2010). The latter include diverse allosteric mechanisms, phosphorylation dependent protein–protein complexation and redox mediated cysteine modification (**Table 1**). Data that emerged over the last two decades suggest that the redox state of the cell plays an important coordinating role in cellular homeostasis by regulation of starch metabolism. Such thiol-based redox mechanisms are more pronounced in eukaryotes than prokaryotes and in also more important in photosynthetic organisms as compared to heterotrophic ones. This suggests an importance of tight link between energy harvest and downstream metabolism in complex autotrophic organisms.

**Table 1:** Redox regulated starch metabolic enzymes.

Enzyme	Organism	Effect on enzyme activity by reduction	Midpoint redox potential (pH 7.0)	Specificity (Trxs and/or NTRC)	Cysteines involved in disulfide formation	Reference
AGPase	Potato	Activation	^∗^n.d.	Trxs *f, m;*	Cys12 of two small subunits in *S. tuberosum*, equivalent to Cys81/82 in *A. thaliana*	Fu et al., 1998; Ballicora et al., 2000; Michalska et al., 2009; Hädrich et al., 2012
	Pea			NTRC		
	Arabidopsis			NTRC		
SS1	Arabidopsis	Activation	−306 mV	Trxs *f1*, *m4*; NTRC	Cys164, Cys545	Skryhan et al., 2015
GWD1	Potato	Activation	−255 mV	Trxs *f, m*	Cys1004, Cys1008	Mikkelsen et al., 2005
SEX4	Arabidopsis	Activation	^∗^n.d.	Trxs *f1, m1, m3, m4, x, y1*	Cys130, Cys198	Silver et al., 2013
BAM1	Arabidopsis	Activation	−302 mV	Trxs *f1, m1, y1;* NTRC	^∗^n.d.	Sparla et al., 2006; Valerio et al., 2011
AMY3	Arabidopsis	Activation	−276 mV	Trxs *f1, m1, m2, m3, m4, x, y1, y2*	Cys499, Cys587	Seung et al., 2013

*^∗^n.d., no data available.*

## Redox-Active Enzymes in Starch Metabolism

A large number of plastidial enzymes involved in carbohydrate metabolism are demonstrated to be redox-sensitive as delineated in **Figure 1**. These data identify enzymes both in the biosynthetic and the degradation pathways, as mainly achieved by using well-defined *in vitro* systems (**Table 1**) and are as such only indicative for a cellular function. However, such mechanistic studies have given important information to identify potential target enzymes, cross-links between photosynthesis and stress-related metabolic steps.

Many of the enzymes demonstrated to be redox sensitive has been thoroughly studied *in vitro* and shown to (i) modulate enzyme activity, (ii) depend on physiological reductants, and (iii) depend on specific protein cysteine(s). Based on these criteria, enzymes of starch metabolism like AGPase, SS1, GWD1, SEX4, BAM1, and AMY3 can be considered redox-regulated. If some of these criteria are not fulfilled, we denote enzymes, including SS3, BE2, ISA1/2/3, BAM3, and LDA, as *suggested* to be redox regulated, implying that further studies *in vitro* and *in vivo*, should be performed to confirm the regulation.

The main identified mechanism involves cystin reversible exchange mediated by thioredoxins (Trxs) having different redox potentials (Yoshida and Hisabori, 2017) and NADP-dependent thioredoxin reductase C (NTRC) leading to conformational change in the target enzyme. Both Trx and NTRC are efficient redox transmitters and for example AGPase and BAM1 are starch-metabolic enzymes known to be reduced by Trx *f*, *m* and NTRC (almost) equally well (Valerio et al., 2011; Thormählen et al., 2013). Typically, target enzymes lose catalytic activity upon oxidation (Couturier et al., 2013) and increased affinity to starch, as for the starch phosphorylator GWD1 (Mikkelsen et al., 2005) and SS1 (Skryhan et al., 2015; **Table 1**).

## Emerging Evidence of Redox Regulation of Transitory Starch Metabolism *In Vivo*

In few cases (AGPase, GWD1), redox regulation was shown also *in vivo* to be influenced by external conditions such as illumination or sugar supply (Hendriks et al., 2003; Kolbe et al., 2005; Hädrich et al., 2012; Skeffington et al., 2014). In most other cases, however, *in vivo* data are completely lacking. This section deals with redox regulation of starch metabolism *in vivo*.

In leaves, AGPase is highly reduced in the light but is increasingly reduced also in the dark when leaves are supplied with sucrose (Hendriks et al., 2003). *In* *vitro*, AGPase is reduced by thioredoxins (mainly *f* and *m*) and by NTRC (Ballicora et al., 2000; Thormählen et al., 2013) and in this way it becomes more sensitive to 3-phosphoglycerate activation (Fu et al., 1998; Ballicora et al., 2000). AGPase reduction, as directly measured in plant extracts, is thus taken as a proxy of its actual activity *in vivo*. Consistently, conditions that lead to AGPase reduction often stimulate also starch accumulation and vice versa, supporting the view that AGPase, in leaves, is reduced/activated in the light by thioredoxins photoreduced by photosystem I (PS-I) via ferredoxin/thioredoxin reductase (FTR), and reduced/activated by sucrose through a different pathway, operative also in the dark, that involves trehalose-6P and NTRC (Kolbe et al., 2005). This view could be too simplistic, but is essentially accepted.

To which extent redox regulation is important for starch metabolism cannot, however, be easily assessed from this kind of experiments. Arguably, reverse genetics provides the more straightforward approaches to address this question. Mutant plants in which the redox regulated cysteine(s) of a redox regulated enzyme are substituted by redox-inactive amino acid residues like serine constitute in principle the best plant material for studying the relevance of redox regulation in a physiological context. To our best knowledge, this approach has been applied only in two cases in starch metabolism, to AGPase (Hädrich et al., 2012) and GWD1 (Skeffington et al., 2014).

AGPase has the analytical advantage that only a single cysteine (Cys-81 in Arabidopsis) of its small subunit APS1 is both necessary and sufficient for its redox regulation, which consists in the formation of a disulfide bridge between two APS1 subunits of the tetrameric enzyme. Mutants with a serine substituting Cys-81 cannot form the disulfide bridge and are permanently redox-activated (Fu et al., 1998; Hädrich et al., 2012). Arabidopsis lines expressing such a mutagenized and permanently active AGPase in a AGPase-knockout background, contain more leaf starch when grown in long-days photoperiods. Interestingly, this was suggested not to be an effect of faster starch synthesis in the light, but slower starch degradation during the night (Hädrich et al., 2012). Hence, a role for AGPase redox regulation in starch turnover was demonstrated, but the metabolic mechanisms are far from clear. Further observations revealed that the mutagenized AGPase, *in vivo*, was degraded faster than the wild type enzyme (Hädrich et al., 2012), suggesting that its physiological turnover was influenced by the redox state of the protein. In wild type plants, oxidized AGPase might be somehow protected from the rapid degradation suffered by mutagenized AGPase, which mimics the reduced form. This hypothesis suggests an additional role of redox regulation in protein turnover (van Wijk, 2015). Such a mechanism is reminiscent of phosphoribulokinase (PRK) that is rapidly degraded in plants in which the three genes of CP12, a protein that redox regulates PRK and GAPDH by assembling a supramolecular complex (Marri et al., 2008, 2009), have been knocked out (López-Calcagno et al., 2017). It has also been shown that oxidized GWD1 from potato (Mikkelsen et al., 2005) and recombinant SS1 from *Arabidopsis thaliana* (Skryhan et al., 2015) have the enhanced affinity for the surface of starch granules. A possible explanation of this phenomenon is that such mechanism is necessary for the enzymes protection under oxidative stress.

GWD1 is the main starch phosphorylator in the plant cell (Blennow and Engelsen, 2010). *In vitro*, GWD1 is completely inactive when disulfide oxidized in the CFATC motif (Mikkelsen et al., 2005). Oxidized GWD tends to bind starch granules in the dark, but a portion of GWD1 remains reduced and soluble in the stroma (Mikkelsen et al., 2005). Interestingly, the redox potential of GWD1 regulatory disulfide (E°’ −250 mV) is much less negative than that of thioredoxin f (E°’−290 mV), suggesting that GWD1 would be reduced *in vivo* under normal conditions (Yoshida et al., 2014). However, granule-bound oxidized GWD1 might be less easily reduced by Trx *f*. The relevance of GWD1 for diurnal starch regulation has been questioned by reverse genetic studies. It turns out that Arabidopsis *gwd* mutants have a strong starch excess phenotype but recover a quasi-normal starch turnover if complemented with, either the wild type, redox-regulated, form or the redox insensitive, mutagenized, GWD1 form (Skeffington et al., 2014). Hence, the physiological role of GWD1 is unclear and it remains to be tested whether the starch granule affinity of the oxidized form (Mikkelsen et al., 2005) can have a protective role, perhaps during severe stress conditions as mentioned above.

Another possibility to test redox regulation *in vivo* is to study mutants of regulatory proteins, especially Trxs, NTRC. However, since both Trxs and NTRC have multiple targets (Cejudo et al., 2012; Pérez-Pérez et al., 2017), results obtained by this approach should be interpreted with caution because of pleiotropic effects. The Arabidopsis genome codes for five classes of plastidial Trxs (*f, m, x, y, z*), each including one (*x, z*) or more isoforms (*f1, f2; m1, m2, m3, m4; y1, y2*) (Meyer et al., 2006). Trxs *m1, m2, m4*, and *f1* are more abundant and, among the 10 isoforms overall, constitute about 90% of the protein content (Okegawa and Motohashi, 2015). *In vitro*, different Trxs have different redox potentials and are reduced by FTR with different efficiency (Yoshida and Hisabori, 2017). *In vivo*, these Trxs are more reduced in the light than in the dark, with differences among isoforms (Yoshida et al., 2014).

Single knockout mutants for Trx *f1* in Arabidopsis, did not show any visible defect in growth or photosynthetic performance, but accumulated less leaf starch and showed a lower activation state of AGPase (Thormählen et al., 2013). Tobacco plants that overexpress Trx *f* conversely accumulate large amounts of starch in chloroplasts, and make more leaf biomass, although the redox state of AGPase was not affected (Sanz-Barrio et al., 2013). Intriguingly, NTRC overexpression also has a positive effect on growth of Arabidopsis plants and accumulated more starch in leaves (Toivola et al., 2013; Nikkanen et al., 2016). NTRC has been demonstrated to promote starch accumulation in response to light or external sucrose treatment via redox-dependent AGPase activation (Michalska et al., 2009). Similar to *trx f1* plants, double *trxf1-f2* mutants also accumulated less starch at the end of the day but, different from *trx f1* plants, also showed more general phenotypic defects including growth retardation in short-day photoperiods and lower photosynthetic transport rates (Naranjo et al., 2016; Ojeda et al., 2017). Low levels of starch were also found in *ntrc* and in *trx x* single mutants (Ojeda et al., 2017), although Arabidopsis AGPase is efficiently activated by NTRC but not as much by Trx *x* (Thormählen et al., 2013). Overall, a scenario seems to emerge in which the level of redox regulatory proteins like Trx *f*, Trx *x*, and NTRC correlate with the amount of transitory starch and, in some cases, with growth. Combinations of double and triple mutants like *ntrc-trx x* and *ntrc-trxf1-f2* confirm the trend since plants impaired in these redox regulatory proteins tend to store less starch in leaves (Ojeda et al., 2017). However, additional effects such as severe growth inhibition, perturbed light acclimation, and impairment of Calvin–Benson cycle activity prevents a simple interpretation of these results.

The complexity of redox regulatory systems was nicely demonstrated by the suppressed growth phenotype of *ntrc* mutants by simultaneous knocking out two 2-Cys peroxiredoxins (2CP, thiol peroxidases involved in antioxidant defense and redox signaling, Pérez-Ruiz et al., 2017). It was proposed that in chloroplasts of wild type plants NTRC is especially involved in reducing 2CP and thus H_2_O_2_, while typical Trxs are reduced by FTR to keep the Calvin–Benson cycle activated in the light. The growth defect of *ntrc* mutant is explained by the capacity of 2CP, in the absence of NTRC, to drain electrons from the Trx pool, thereby causing an indirect downregulation of the Calvin–Benson cycle. Abolishing the electron withdrawal by additional knocking out the 2CPs restores the capacity of FTR-reduced Trxs to activate the Calvin–Benson cycle and thereby growth (Pérez-Ruiz et al., 2017). Clearly, phenotypes of knockout mutants of redox regulatory proteins (like *ntrc*) must be interpreted with care.

## Transitory Starch Re-Cycling: the Current Debate on Simultaneous Biosynthesis and Degradation of Starch in the Light

In the last two decades the pathway of transitory starch breakdown has been deeply detailed (Yu et al., 2001; Ritte et al., 2002; Niittylä et al., 2004; Fulton et al., 2008; Kötting et al., 2009). The widely accepted model describes a night-active pathway for starch degradation to balance the lack of triose phosphates from the Calvin–Benson cycle. Accordingly, starch behaves as a carbon buffer to fuel plant metabolism and growth when photosynthesis is inactive (Zeeman et al., 2010; Stitt and Zeeman, 2012). However, there is support for the existence of starch degradation in illuminated leaves both in the absence (Stitt and Heldt, 1981; Baslam et al., 2017; Fernandez et al., 2017) and in the presence (Valerio et al., 2011; Feike et al., 2016; Thalmann et al., 2016; Zanella et al., 2016) of stress.

There is a general agreement that stresses ranging from increased photorespiratory rate to more severe osmotic stress, trigger leaf starch degradation in light (Lu et al., 2005; Weise et al., 2006; Valerio et al., 2011; Prasch et al., 2015; Thalmann et al., 2016; Zanella et al., 2016). In relation to redox regulation, it is worth mentioning that degradative enzymes can also be reductively activated. At first sight, reductive activation of enzymes involved in starch degradation is counterintuitive since such degradation would interfere with active starch accumulation during the day and loss of activity during the night-time. Nevertheless, starch degradation has actually been shown to take place simultaneously with starch synthesis under long day conditions (Fernandez et al., 2017). Starch degradation during the day can also play a physiological role under certain stress conditions as demonstrated for *At*BAM1 being active upon osmotic stress (Valerio et al., 2011) and *At*AMY3 showing increased expression after cold shock. Additionally, starch accumulation was elevated in mutants lacking AMY3 (Seung et al., 2013). Another option is a spatial separation of starch degradation which was demonstrated for guard cells where starch degradation by the BAM1 sustains stomata opening (Valerio et al., 2011; Santelia et al., 2015). Ability of some redox-sensitive targets to be activated by NTRC, which takes the reducing power from the light-independent oxidative pentose phosphate pathway, can provide a reductive activation of starch degrading enzymes in the night.

To what extent diurnal starch degradation contributes to starch turnover in the light is still debated. Two very recent studies have demonstrated active diurnal starch degradation in plants exposed to continuous light (Baslam et al., 2017; Fernandez et al., 2017). One study (Fernandez et al., 2017) proposes that diurnal starch degradation only occurs late in the day (over 14 h from dawn) following the classical pathway illustrated in **Figure 1**. A second model (Baslam et al., 2017) proposes extensive starch degradation in the light based on a carbon cycle around ADP-glucose (Baroja-Fernández et al., 2004; Baslam et al., 2017). Accordingly, export of triose phosphate to the cytosol would result in the production of ADP-glucose through the activity of sucrose synthase (Baroja-Fernández et al., 2004; Baslam et al., 2017) that could enter the chloroplast (Pozueta-Romero et al., 1991), be converted into starch, and be degraded to sustain plant growth even in the light (Baslam et al., 2017). Although intriguing, the main obstacle to this model is that the role of chloroplastic AGPase in the synthesis of starch would be marginal and the strong defective phenotype of the AGPase mutant difficult to explain (Lin et al., 1988a,b; Wang et al., 1997, 1998).

## Future Directions

Although significant advances in the understanding of redox regulation of starch metabolism have been made, many questions still remain open. Especially, much research has been conducted on the redox response of chloroplastic enzymes since transitory starch is a major product of leaf photosynthesis through the Calvin–Benson cycle suggested to be strictly redox-regulated (Michelet et al., 2013). Transitory starch metabolism changes dramatically under light or dark conditions, and this behavior may link diel starch metabolism to the light-dependent redox state of chloroplast thioredoxins *in vivo* (Yoshida et al., 2014). Emerging evidence supports the view that starch accumulation in illuminated leaves is positively correlated with the reduced state of the starch biosynthetic enzyme AGPase and, in general, with the capacity of the redox regulatory machinery (see section “Emerging Evidence of Redox Regulation of Transitory Starch Metabolism *in vivo*”). However, evidence for the physiological relevance of the redox sensitivity of many starch metabolic enzymes is still incomplete and stronger biochemical evidence is required.

*In vivo* studies have clearly demonstrated the relevance of redox regulation for starch metabolism through reverse genetic approaches on redox regulatory proteins (*trx f1*, Thormählen et al., 2013; *trx f1-f2*, Naranjo et al., 2016; *ntrc* and *trx x*; Ojeda et al., 2017). Although the analysis of knock out mutants has greatly contributed to the discovery of starch synthesis and degradation pathways, this approach is limited since thioredoxins and NTRC have several targets which complicates the interpretation of data.

Mutating specific cysteines responsible for the redox regulation of metabolic enzymes appears as a more promising approach to directly deduce the mechanisms of regulation (Hädrich et al., 2012; Skeffington et al., 2014; Skryhan et al., 2015). The introduction of genome editing opens an exciting scenario since this method allows direct modification of genes of interest, avoiding additional genetic variations. However, any attempt to precisely modify a DNA coding sequence *in vivo* will require deeper biochemical *in vitro* knowledge of the structure and behavior of the coded protein.

Hence, combinatorial approaches would be required shed new light on the importance of redox regulation in starch metabolism. Such information is urgently required considering that starch is fundamental component of food, feed, and future materials like bioplastics. Efficient agro-production to feed the doubling world population in 2050 and to solve fundamental environmental issues like plastics pollution requires stress-tolerant and robust starch crops. Controlling redox modulation of starch crops is a central point for maximizing crop efficiency in a future changing climate.

## Author Contributions

All authors contributed equally to the content of this paper.

## Conflict of Interest Statement

The authors declare that the research was conducted in the absence of any commercial or financial relationships that could be construed as a potential conflict of interest.
